# Association of physical fitness with quality of life in community-dwelling older adults aged 80 and over in Poland: a cross-sectional study

**DOI:** 10.1186/s12877-021-02421-5

**Published:** 2021-09-09

**Authors:** Ewelina Lepsy, Ewa Radwańska, Grzegorz Żurek, Alina Żurek, Antonina Kaczorowska, Alina Radajewska, Anna Kołcz

**Affiliations:** 1 Institute of Health Sciences, University of Opole, Opole, Poland; 2 Department of Biostructure, University School of Physical Education, Wroclaw, Poland; 3 Department of Clinical Psychology and Health, University of Wroclaw, Wroclaw, Poland; 4 Department of Physiotherapy, Krapkowice Health Centre, Krapkowice, Poland; 5 Laboratory of Ergonomics and Biomedical Monitoring, Wroclaw Medical University, Grunwaldzka 2, 50-355, Wroclaw, Poland

**Keywords:** Quality of life, Physical fitness, Psychosocial factors, Lifestyle

## Abstract

**Background:**

Aging is a progressive and irreversible process that negatively affects the quality of life (QOL). Older adults face difficulties related to worsening health, lowering the level of physical and mental efficiency. We aimed to analyze the associations between physical fitness and QOL in Polish older adults considering sex differences.

**Methods:**

This cross-sectional study was performed from March to August 2015. The sample consisted of 100 community-dwelling adults (67 women, 33 men) with a mean age of 82.94 ± 2.67 years. The World Health Organization QOL, Short Form questionnaire (WHOQOL-BREF), and the Fullerton Functional Fitness Test (FFFT) were used. Biometric data, social and environmental situation, nutritional and lifestyle behaviors have been also collected using a questionnaire designed by the authors.

**Results:**

The results obtained in individual domains of WHOWOL-BREF indicate a good level of QOL in all the examined domains. Statistically significant sex differences were obtained in physical (*p* = 0.01), psychological (*p* = 0.04) and environmental (*p* = 0.02) domains in WHOQOL-BREF. It was noted that men perform better in terms of the upper (arm curl, *p* < 0.001) and lower body strength (chair stand, *p* = 0.01), aerobic endurance (two-minute step test, *p* < 0.001), agility and dynamic balance (up and go test, *p* < 0.001) in FFFT.

**Conclusions:**

Community-dwelling older adults aged 80–93 years in Poland present a good level of QOL, and the higher score was obtained in men. Also, men presented better physical fitness, showed a higher level of independence in daily activities, and assessed better their own QOL than women.

## Background

The reflection on aging should begin with an awareness of the scale of this phenomenon, which affects all aspects of life. Excessive aging is noteworthy, relating to the increase in the proportion of the elderly population, a subpopulation of very older people (80 years of age and above) [1]. This phenomenon results from two well-known reasons for the aging of the population – the first one is a decline in the number of births and the second one is a lower mortality rate, as confirmed by the most recent global decomposition analysis [2].

According to current EUROSTAT data [3], the European Union is an agglomeration of more than 500 million people, 19.2% (approximately 100 million) are older people. Demographic data predict that the number of people aged 80 and over will double in European countries by 2060. In Germany, Slovakia, Spain, Portugal, and Greece, the percentage will be 13.4–16.1% of the total population and Poland 12.3% [4, 5]. The aging population causes dramatic demographic, epidemiological, and anthropological changes, emphasizing active, and healthy aging philosophy [6].

According to the World Health Organization (WHO) [7], planning an exercise training program for older people should consider their goals and aspirations to be more motivated to engage in systematic physical activity. One of the most important factors influencing the health condition of the elderly is physical activity [8, 9].

Systematically undertaken physical activity supports the treatment of chronic diseases and enables a healthy, active life without functional barriers [10–12]. Moreover, the reduction of physical activity in older people promotes many chronic diseases, including hypertension, diabetes, obesity, cardiovascular diseases, strokes, and some cancers [13, 14]. Recent studies highlighted that systematic and appropriately dosed physical activity could delay aging processes [15, 16]. It also allows older people to maintain their physical fitness at a level that enables them to function more independently [17, 18]. Evidence from observational studies supports the beneficial effects of physical activity on cognition [19, 20]. However, strong evidence from randomized controlled trials is still lacking, and e.g., in the LIFE study, beneficial effects of physical activity on cognition were only seen in subgroup analyses [21, 22].

As life expectancy is still increasing among the world’s population, the main concern is whether the extended time is related to years of healthy life and promotes a high health-related quality of life (HRQOL) until old age [23]. Regular physical activity, which helps to improve physical and mental functions and reverse some of the effects of chronic diseases to maintain the mobility and independence of older people, will become increasingly important [24]. Physical fitness and physical activity play an essential role in the quality of life (QOL) of older adults, especially in those who often have difficulties in everyday activities and psychological and social functioning [25].

Daimiel et al. [26], in their PREDIMED-Plus trial, showed that higher levels of physical activity and physical fitness are strongly associated with a better level of QOL – higher scores in all domains of the SF36-HRQL questionnaire. Polish cross-sectional study by Puciato et al. [27] conducted among over 1000 participants showed that the overall QOL, the perceived health status, and the QOL in the physical, psychological, social, and environmental domains of WHOQOL-BREF were significantly better in people with higher levels of physical activity. Also, Oh et al. [28] investigated the effects of three of the most representative exercises (resistance, flexibility, and walking) on QOL in a population of community-dwelling older adults. They observed that QOL parameters (EuroQOL) such as mobility, self-care, usual activities, pain/discomfort, anxiety/depression were improved.

Numerous studies have been undertaken on QOL and activity in older people [29, 30] as well as age-related physical disability [31, 32]. In Poland, it was found that in population of people aged 60–80, almost 30% suffer from at least a moderate level of disability and over 10% experience severe disability [33]. However, the dominant age group studied were people between 60 and 80 years of age. As the number of people aged 80 and over is growing, there is a need to acquire up-to-date knowledge to improve the QOL. For this age group, there is a lack of studies on the impact of involutional processes that consider the level of independence in everyday life, lifestyle, activity, and physical fitness on the assessment of QOL.

The study aimed to analyze the association between physical fitness level with the QOL of Polish older adults. For this purpose, two main research questions were formulated: (1) Are the general QOL and its particular domains (physical, psychological, and social) associated with the level of physical fitness among the studied population? (2) Are there any sex differences in the relationship between physical fitness and QOL among the studied population? It was assumed that higher status of chosen physical, psychological, and social domains is related with better level of physical fitness as well as that men present better physical fitness which is associated with their higher QOL than in women.

## Methods

### Study design and participants

This cross-sectional study was conducted in south-western Poland (Opole, Lower Silesia, and Silesia) from March to August 2015. The study consisted of 100 community-dwelling adults (67 women and 33 men) aged 80–93. The STROBE (STrengthening the Reporting of OBservational studies in Epidemiology) guidelines for observational studies were followed.

### Qualification criteria

The criteria for inclusion covered persons who: (1) were aged 80 years and above at the start of the study, (2) did not show any impairment in their ability to respond logically and independently, (3) lived in an independent or shared household, and (4) gave informed and voluntary consent to participate in the study. In turn, the exclusion criteria were: (1) health factors that make it impossible to conduct the study, such as consciousness disorders, psychosis, dementia, cognitive disorders, (2) distrust, aversion, and apparent fear of the test subjects towards the test subject’s home visit, (3) living in centers providing care for the elderly, and (4) lack of consent to participate in the study.

### Data collection

The study participants were recruited based on eligibility data from rehabilitation facilities in three districts of south-western Poland, and the target population was older individuals aged over 80 years. The survey was conducted individually at the home of the examined person after obtaining voluntary consent. The home visits took place in the morning, when the psychophysical condition of the respondents was the most favorable. Before the study began, the participants were informed about the purpose of the study, and they were instructed on how to answer the questions. During the survey, everyone could obtain additional information in case of any ambiguities. Each survey lasted from 1.5 to 2 h and was started with a survey-based interview, always in a specific procedure. Subsequently, questions were asked according to an original questionnaire (Appendix 1).

The received answers and statements were noted during the home visit of the interviewers. The survey investigators took part in the training, during which they were familiarized with the objectives of the study, the structure of the questionnaires, and instructions on how to fill them out and how to communicate effectively with the respondents. All participants were informed about the purpose and course of the study and agreed in writing to participate. During the study, we attempted to contact 150 individuals anticipated to participate in the study, of which 123 were successfully contacted. A final sample of 100 individuals was obtained during qualification. Our contact rate of 68.33% and response rate of 81.30% are reasonable and consistent with response rates obtained in other population-based studies.

### Sample size

The estimated sample size for a two-sample unpaired-means test (unpaired t-test) was calculated using Statistica 12 (TIBICO, Inc., USA). For the Student’s t-test, the test power for body height was 1.000, and body mass was 0.997. The minimum number of samples is N1 = N2 = 20 (for height) and N1 = N2 = 25 for body mass. The alpha level was set at 0.05, and the power of the test 1-beta at 0.9. It also assumed no correlation of evaluated variables and adopted a 2-sided null hypothesis. Based on the parameters, the estimated sample size has been obtained for a minimum number of patients in both compared groups is N1 (women) = N2 (men) = 25 in the study. The final sample size in this research was 100 participants.

### Measurements

The following research tools were used to assess study outcomes: (1) World Health Organization QOL Standardized Questionnaire, Short Form (WHOQOL-BREF) to assess QOL levels, (2) Fullerton Functional Fitness Test (FFFT) to assess physical performance, and (3) original survey questionnaire designed for the purpose of this study including 40 questions on biometric data, anthropometric characteristics, family and environmental situation, nutritional and lifestyle behaviors (Appendix 1).

#### World Health Organization QOL, short form (WHOQOL-BREF)

The QOL examination of older adults was performed using the WHOQOL-BREF questionnaire based on the WHOQOL 100; it is designed for subjective QOL assessment [34]. It analyses four primary areas of life: physical, psychological, social, and environmental, as well as overall QOL and self-assessment of health. In the physical field, older adults are assessed: activities of daily living, dependence on medicinal substances and medical aids, energy and fatigue, mobility pain and discomfort, sleep and rest, and work capacity. In the psychological field: bodily image and appearance, negative feelings, positive feelings, self-esteem, spirituality/religion / personal beliefs, thinking, learning, memory, and concentration. In the social field: personal relationships, social support, sexual activity. In the environmental field: financial resources, freedom, physical safety and security, health and social care (accessibility and quality), home environment, opportunities for acquiring new information and skills, participation in and opportunities for recreation/leisure activities physical environment, (pollution, noise, traffic, climate), and transport [35].

The WHOQOL-BREF questionnaire is used to evaluate the psychometric QOL; on a five-stage scale, the respondent’s emotional and physical state is analyzed. The score for particular domains is determined by calculating the arithmetic mean from the positions included in particular domains. The scoring has a positive direction, which means that more points indicate a better QOL [36]. According to Jaracz et al. [37], who described psychometric properties of the Polish WHOQOL-BREF, it was shown a high validity ranged between 0.62–0.76 for the physical domain, 0.55–0.78 for the psychological domain, 0.68–0.85 for the social domain, and 0.58–0.68 for the environmental domain.

#### Fullerton functional fitness test (FFFT)

The FFFT tool, based on the American College of Sport Medicine, was used to assess physical fitness using several medical consultations. It was published by Rikli and Jones [38] and assesses all the physiological properties that are necessary to maintain safe daily activity and independence. It is designed to assess the functional performance of older adults, i.e., those over 60 years of age, and consists of six trials to assess the strength of the upper body – arm curl; the strength of the lower body – chair stand; flexibility within the upper body – back scratch test; flexibility within the lower body – chair sit-and-reach; agility and dynamic balance – 8-ft up and go; aerobic endurance – six-minute walk test or two-min step test.

Instruction and a demonstration preceded the performance of tests. In Polish conditions, the adaptation of units of measurement and weight (e.g., inches, feet, pounds) was used, making it possible to perform tests using commonly available tools. The FFFT is a safe research tool for the elderly and can be used without additional medical examinations. It is easy to perform and requires no special equipment. It is a useful research tool because it allows identifying areas of individual weakness and preparing intervention programs, and comparing the results of individuals of the same age and sex [39]. The FFT represents good reliability and variability ranged between 0.79–0.97, and the repeatability between 0.80–0.97 [39]. This measurement tool was also used in the previous studies among polish population by Ignasiak et al. [40], Umiastowska and Kupczyk [41], and Nawrocka et al. [42].

### Ethical considerations

The study protocol was approved by the Bioethics Committee of the Opole Medical School, Poland (permission no. 3/2015). All patients provided informed consent and were informed that they could withdraw from the study at any stage. The study was carried out following the tenets of the Declaration of Helsinki and Good Clinical Practice guidelines.

### Statistical analyses

The results were analyzed using Statistica 12 (TIBICO, Inc., USA). All quantitative variables were tested with the Shapiro-Wilk test to determine the type of distribution. For parameters where there were no grounds for rejecting the normal distribution hypothesis, mean values (M), standard deviation (SD), and coefficient of variation (*v*) were calculated. For parameters where the normal distribution hypothesis was rejected, median (Me), lower (Q1), and upper (Q3) quartiles, minimum (min), and maximum (max) were calculated. The significance of differences between the mean values was estimated using the Student’s t-test. Differences between the groups were calculated with the Mann-Whitney U test. The Pearson correlation coefficient (for normal distribution) and Spearman’s ranked correlation coefficient (for non-normal distribution) were used. In order to assess the relationship between a dependent variable and a collection of independent variables, multiple regression analysis was applied (step regression). In all analyses, the level of *p* < 0.05 was assumed to be statistically significant.

## Results

The study group included women (*n* = 67) and men (*n* = 33) aged 80–93 years. The major group was people living in the countryside (76%), in 82% with primary vocational education. The characteristic of the study group is presented in Table 1.

**Table 1:** Characteristics of study participants including sex differences (*n* = 100)

Feature	Women (*N* = 67)		Men (*N* = 33)		Student’s t-test		
	M	SD	M	SD	t	p	1-β
Age [years]	82.9	2.7	83.0	3.4	−0.14	0.887	
Height [cm]	157.9	5.5	170.1	4.8	−10.76	**< 0.001**	**1.000**
Body mass [kg]	68.5	9.9	77.4	8.3	−4.43	**< 0.001**	**0.994**
BMI [kg/m^2^]	27.46	3.85	26.77	2.77	0.92	0.358	

*Abbreviations*: *BMI* body mass index, *N* number of participants, *M* mean, *SD* standard deviation, *t* t-quantile, *p* statistical significance, *1-β* power of the test

*Notes*: *values in bold indicate statistical significance (*p* < 0.05)

The first two items of WHOQOL-BREF (overall QOL and general health) were analyzed separately and concerned individual perception of QOL. The mean values of the overall QOL and general health in men were slightly higher than in women. The mean values of individual general health were significantly different (Table 2).

**Table 2:** Between sex differences for comparisons in QOL domains and physical fitness parameters

Feature	Women (*N* = 67)		Men (*N* = 33)		Student’s t-test	
	M	SD	M	SD	t	p*
Overall QOL [score]	3.81	0.94	4.09	0.81	−1.49	0.1393
General health [score]	3.42	1.02	3.85	0.71	−2.18	**0.0317**
Physical domain [score]	24.27	5.62	27.24	4.6	−2.63	**0.0099**
Psychological domain [score]	22.84	4.55	24.73	3.67	−2.08	**0.0404**
Social domain [score]	11.67	1.85	11.88	1.88	−0.52	0.6024
Environmental domain [score]	31.08	4.81	33.52	4.44	−2.45	**0.0162**
Chair stand [n]	7.34	3.33	9.55	4.35	−2.8	**0.0061**
Arm curl [n]	9.78	3.94	12.97	4.83	−3.53	**0.0006**
Two-minute step test [n]	26.88	18.49	39.24	19.96	−3.06	**0.0028**
Back scratch test [cm]	−22.71	17.6	−26.45	20.12	0.94	0.349
Up and go [s]	19.93	12.64	12.03	4	3.49	**0.0007**
Feature	Women (*N* = 67)		Men (*N* = 33)		Mann-Whitney U test	
	Me	Min-Max	Me	Min-Max	z	p*
Vertical jump test [cm]	0	−49–5	0	−36–8	0.43	0.6873

*Abbreviations N* number of participants, *QOL* quality of life, *M* mean, *SD* standard deviation, Me median, *Min* minimum, *Max* maximum, *t* t-quantile, *p* statistical significance, *z* z-quantile

*Notes*: *values in bold indicate statistical significance (*p* < 0.05)

Furthermore, a much better perception of environmental, psychological, and physical functioning was shown in men. In turn, the highest scores in social relations and environmental functioning were observed in women. In both groups, the lowest score was in the physical domain. The evaluation of social relations was almost the same in women and men. The analysis showed statistically significant differences between the mean scores in physical, psychological, and environmental domains (Table 2).

In the FFFT, statistically significant sex differences in the mean values in chair stand, arm curl, two-minute step test, and up and go was shown in favor of men. In the back scratch test, better results were achieved by women; however, not statistically significant (Table 2).

Also, a correlation was shown between the overall QOL and the results of the FFFT in older people (Table 3). The most significant relationships in women were observed between the overall QOL, general health, and all domains of WHOQOL-BREF and the results of the four FFFT tests (chair stand, arm curl, two-minute step test, and up and go). It was observed that the higher the physical fitness, the higher the QOL in women. In the group of men, there were less significant relationships observed. The environmental domain was correlated with the four FFFT tests (chair stand, arm curl, two-minute step test, and sit and reach test). The physical domain was correlated with the results of three FFFT test (up and go, arm curl, chair sit-and-reach) and the psychological domain with the results of two FFFT test (up and go, and 2-min walk) (Table 3).

**Table 3:** Between sex differences for correlations of the QOL domains and physical fitness parameters

	Feature	Age [years]	Height [cm]	Body mass [kg]	BMI [kg/m^2^]	Chair stand [n]	Arm curl [n]	Two-minute step test [n]	Sit and reach test [cm]	Back scratch test [cm]	Up and go [s]
Women (*N* = 67)	Overall QOL [score]	−0.12	−0.20	− 0.08	0.01	**0.50**	**0.40**	**0.47**	0.07	0.13	**−0.53**
	General health [score]	−0.16	−0.17	− 0.19	−0.06	**0.60**	**0.34**	**0.44**	0.15	0.09	**−0.61**
	Physical domain	−0.26	−0.13	− 0.02	0.08	**0.56**	**0.39**	**0.37**	0.19	0.08	**−0.58**
	Psychological domain	−0.28	0.14	−0.05	0.04	**0.57**	**0.33**	**0.48**	0.12	0.12	**−0.56**
	Social domain	−0.16	−0.14	0.03	0.09	**0.41**	0.17	**0.38**	0.12	0.10	**−0.43**
	Environmental domain	−0.08	−0.16	− 0.03	0.03	**0.45**	**0.37**	**0.31**	0.09	0.20	**−0.48**
Men (*N* = 33)	Overall QOL [score]	0.14	−0.14	−0.05	− 0.04	0.21	0.13	0.42	0.23	0.11	−0.12
	General health [score]	−0.05	−0.11	0.01	−0.01	0.21	0.13	0.26	0.07	0.14	0.09
	Physical domain	0.06	−0.21	0.04	0.09	**0.48**	**0.37**	**0.47**	**0.46**	0.06	−0.25
	Psychological domain	−0.02	−0.04	0.15	0.16	**0.46**	0.32	**0.53**	0.26	−0.11	−0.28
	Social domain	−0.08	−0.15	− 0.03	0.03	0.15	0.28	0.15	0.19	0.17	−0.02
	Environmental domain	−0.14	−0.16	0.24	0.27	**0.55**	**0.51**	**0.47**	**0.43**	−0.11	−0.31

*Abbreviations*: *N* number of participants, *QOL* quality of life, *BMI* body mass index

*Notes*: *values in bold indicate statistical significance (*p* < 0.05)

A correlation was shown between women’s age and the results of three FFFT test (chair stand, two-minute step test, and back scratch test). A different perception of the physical domain with age was presented in women than men. The psychological and social domains decrease with age in the group of women and men, but it shows a stronger tendency in the group of women (Table 4).

**Table 4:** Between sex differences for comparisons of QOL domains and physical fitness parameters according to participants’ age

Feature		Effect							
		b			Age [years]				
		Param.	t	p*	Param.	t	p*	β	SE β
Women (*N* = 67)	Height [cm]	134.1	6.03	**< 0.001**	0.287	1.07	0.288	0.137	0.128
	Body mass [kg]	72.2	1.86	0.068	−0.038	− 0.08	0.935	− 0.011	0.129
	BMI [kg/m^2^]	35.64	2.38	**0.020**	−0.096	−0.53	0.595	−0.069	0.129
	Chair stand [n]	34.70	2.70	**0.009**	−0.330	−2.13	**0.038**	−0.265	0.124
	Arm curl [n]	22.00	1.39	0.168	−0.147	− 0.78	0.441	− 0.100	0.128
	Two-minute step test [n]	191.17	2.80	**0.007**	−1.979	−2.40	**0.019**	−0.296	0.123
	Sit and reach test [cm]	68.54	1.62	0.110	−0.886	−1.74	0.087	−0.219	0.126
	Back scratch test [cm]	135.40	2.02	**0.048**	−1.911	−2.37	**0.021**	−0.292	0.123
	Up and go [s]	−22.37	−0.47	0.642	0.501	0.87	0.389	0.111	0.128
	Overall QOL [score]	7.32	1.95	0.056	−0.042	−0.94	0.351	−0.121	0.128
	General health [score]	8.62	2.19	**0.032**	−0.063	−1.33	0.188	−0.169	0.127
	Physical domain	71.12	3.37	**0.001**	−0.563	−2.21	**0.031**	−0.275	0.124
	Psychological domain	65.34	3.90	**< 0.001**	−0.512	−2.53	**0.014**	−0.311	0.123
	Social domain	20.92	2.88	**0.006**	−0.111	−1.27	0.208	−0.162	0.127
	Environmental domain	44.02	2.39	**0.020**	−0.154	− 0.70	0.490	− 0.089	0.129
Men (*N* = 33)	Height [cm]	207.9	10.03	**< 0.001**	−0.456	−1.83	0.078	−0.327	0.179
	Body mass [kg]	128.1	3.52	**0.002**	−0.602	−1.37	0.180	−0.251	0.183
	BMI [kg/m^2^]	31.44	2.53	**0.017**	−0.053	−0.36	0.724	−0.067	0.189
	Chair stand [n]	24.24	1.31	0.201	−0.175	−0.79	0.438	−0.147	0.187
	Arm curl [n]	21.28	1.11	0.278	−0.096	−0.41	0.683	−0.078	0.188
	Two-minute step test [n]	20.51	0.23	0.818	0.242	0.23	0.821	0.043	0.189
	Sit and reach test [cm]	11.49	0.44	0.665	−0.175	−0.55	0.584	−0.104	0.188
	Back scratch test [cm]	−32.74	−0.36	0.724	0.068	0.06	0.952	0.012	0.189
	Up and go [s]	39.19	2.28	**0.030**	−0.329	−1.59	0.122	−0.289	0.181
	Overall QOL [score]	0.41	0.11	0.912	0.044	1.00	0.327	0.185	0.186
	General health [score]	4.87	1.55	0.133	−0.013	−0.33	0.743	−0.062	0.189
	Physical domain	20.76	1.04	0.309	0.082	0.34	0.735	0.064	0.189
	Psychological domain	38.76	2.34	**0.027**	−0.169	−0.85	0.405	−0.158	0.187
	Social domain	22.09	2.62	**0.014**	−0.124	−1.22	0.232	−0.225	0.184
	Environmental domain	62.43	3.42	**0.002**	−0.345	−1.57	0.127	−0.285	0.181

*Abbreviations*: *BMI* body mass index, *QOL* quality of life, *b* regression coefficient, *SE* standard error, *t* t-quantile, *p* statistical significance, *β* standardized regression coefficient

*Notes*: *values in bold indicate statistical significance (*p* < 0.05)

Required Sample Size: N1 = N2 = 26

In the group of women, the environmental domain was related to the results of two FFFT test (arm curl, and up and go); however, chair stand was associated with the remaining physical, psychological, and social domains. The range values of ß standardized for four domains in women were from − 0.34 to 0.57. The performance of up and go test was shown a negative correlation (Table 5).

**Table 5:** Regression analysis of particular QOL domains according to physical fitness parameters among women

Effect		b	SE b	t	p*	β	SE β
Physical domain	b	2.508	0.204	12.31	**< 0.001**		
	Chair stand [n]	0.132	0.025	5.19	**< 0.001**	0.544	0.105
	Arm curl [n]						
	Two-minute step test [n]						
	Back scratch test [cm]						
	Up and go [s]						
Psychological domain	b	2.845	0.187	15.17	**< 0.001**		
	Chair stand [n]	0.131	0.023	5.62	**< 0.001**	0.575	0.102
	Arm curl [n]						
	Two-minute step test [n]						
	Back scratch test [cm]						
	Up and go [s]						
Social domain	b	3.296	0.168	19.67	**< 0.001**		
	Chair stand [n]	0.082	0.021	3.92	**0.0002**	0.440	0.112
	Arm curl [n]						
	Two-minute step test [n]						
	Back scratch test [cm]						
	Up and go [s]						
Environmental domain	b	3.780	0.245	15.44	**< 0.001**		
	Chair stand [n]						
	Arm curl [n]	0.044	0.018	2.49	**0.0152**	0.288	0.115
	Two-minute step test [n]						
	Back scratch test [cm]						
	Up and go [s]	−0.016	0.005	−2.99	**0.0039**	−0.346	0.115

*Abbreviations*: *b* regression coefficient, *SE* standard error, *t* t-quantile, *p* statistical significance, *β* standardized regression coefficient

*Notes*: *values in bold indicate statistical significance (*p* < 0.05); required sample size: N1 = N2 = 25

In the group of men, significant correlations were shown with the results of two FFFT test (2-min walk, and chair stand0; however, the results of the two-minute step test were associated significantly with physical and psychological domains. The environmental domain was related to the results of the chair stand test. The range value of ß standardized for these three domains was from 0.47 to 0.53. In the men studied, no fitness test was significantly related to the social domain QOL (Table 6).

**Table 6:** Regression analysis of particular QOL domains according to physical fitness parameters among men

Effect		b	SE b	t	p*	β	SE β
Physical domain	b	3.315	0.229	14.49	**< 0.001**		
	Chair stand [n]						
	Arm curl [n]						
	Two-minute step test [n]	0.015	0.005	2.97	**0.0058**	0.477	0.160
	Back scratch test [cm]						
	Up and go [s]						
Psychological domain	b	3.534	0.220	16.06	**< 0.001**		
	Chair stand [n]						
	Arm curl [n]						
	Two-minute step test [n]	0.015	0.005	3.02	**0.0051**	0.483	0.160
	Back scratch test [cm]						
	Up and go [s]						
Social domain	b	3.969	0.112	35.32	**< 0.001**		
	Chair stand [n]						
	Arm curl [n]						
	Two-minute step test [n]						
	Back scratch test [cm]						
	Up and go [s]						
Environmental domain	b	3.507	0.217	16.17	**< 0.001**		
	Chair stand [n]	0.071	0.020	3.46	**0.0017**	0.534	0.154
	Arm curl [n]						
	Two-minute step test [n]						
	Back scratch test [cm]						
	Up and go [s]						

*Abbreviations*: *b* regression coefficient, *SE* standard error, *t* t-quantile, *p* statistical significance, *β* standardized regression coefficient

*Notes*: *values in bold indicate statistical significance (*p* < 0.05); required sample size: N1 = N2 = 25

Mobility endurance, upper and lower body strength, balance, coordination, and speed are associated with the particular domains of QOL among older adults studied.

## Discussion

Our study indicates that community-dwelling older adults aged 80–93 years in Poland present a good level of QOL, which is associated with a good level of physical fitness. However, a higher level of QOL was observed in men. Similarly, men present better physical fitness and physical activity, including mobility endurance, upper and lower body strength, balance, coordination, and speed. Moreover, men show a higher level of independence in daily activities and assess their QOL better than women. It was noted that men had better results in FFFT in terms of the upper (arm curl) and lower body strength (chair stand), aerobic endurance (two-minute step test), agility, and dynamic balance (up and go test). It was also observed that the level of physical fitness of women decreased more with age than in men. In the upper-body flexibility test (back scratch test), the women showed a better range of motion in the upper limb joints.

The last stage was a step-by-step regression analysis for the parametric results of the five performance tests: chair stand, arm curl, two-minute step test, back scratch test, and up and go test. These variables best explain the environmental, physical, and psychological QOL variables in both studied groups. The most dominant sphere of QOL of older adults in old age is the environmental sphere. This sense of security, health care, good material, living conditions, access to information, and realization of interests play a crucial role in assessing QOL.

Poland lacks specific standards for assessing the level of physical fitness of older people, particularly those over 80. Our study results can be very carefully related to the standards developed in the USA, in which 7183 people were tested, including 5048 women and 2135 men in day-care center residents [43]. In such a comparison, the Polish people aged 80 and above have been disadvantageous concerning American older adults. Particularly in the “two-minute step” and “up and go” tests, the results in both examined groups were significantly below the lower limit of the norm; only in the strength of the upper and lower body parts Polish older adults reached the lower limit for a comparable age group of US residents. On the other hand, in tests assessing upper and lower rim flexibility, the women surveyed achieved quite similar results to American standards, while the results of men, especially regarding lower body flexibility, were much lower.

Similar international research to ours has been conducted by Ignasiak et al. [44, 45], Grześkowiak et al. [46], and Katan et al. [47] in women and men, but younger age groups (50 to 76 years). In these studies, the results were also much worse than in the American population. The differences in the results obtained in our study can be considered due to the low level of physical activity of Polis older adults, as pointed out by Bień [48] as early as 2001. At that time, 80% of people over 75 years old did not need to participate in rehabilitation programs.

The results of our study correspond with the results of the POLSENIOR project; the percentage of women with good QOL is lower than men [49]. Men rated QOL better than women in physical, psychological, and environmental fields. The self-assessment of health in our study in both groups was satisfactory, similarly, in the study by Waszkiewicz et al. [50]. The percentage of people satisfied with their health was higher among people over 80. Analyzing the QOL profile in the scope of individual domains of the WHOQOL-BREF questionnaire, the highest scores were obtained in the group of men in the environmental and psychological spheres, in women in the environmental and social relations. Chruściel et al. [51] showed that living with older adults’ relatives may be expected to be favorable for older adults because it translates into better physical, psychological and social domains. Also, loneliness, which frequently accompanies old age, leads to the deterioration of the QOL [51–53].

Polish cross-sectional study by Puciato et al. [27] conducted among over 1000 participants with the use of WHOQOL-BREF showed that the overall QOL, the perceived health status, and the QOL in the physical, psychological, social, and environmental domains were significantly better in people with higher levels of physical activity assessed with the use of the International Physical Activity Questionnaire Short Version (IPAQ-SF). The highest mean indicators of overall QOL perceived health status and QOL in the physical, psychological, social, and environmental domains were shown in the older adults with the highest physical activity, which corresponded to our results.

Also, Nawrocka et al. [42] demonstrated that physical activity level is significantly associated with the social relationships domain of QOL. The researchers identified the differences in functional fitness (Senior Fitness Test and hand-grip strength) and QOL (WHOQOL-BREF) among women over 60 years of age depending on their level of objectively measured physical activity according to the Global Recommendations on Physical Activity for health. It was demonstrated a significant association between the upper body strength, dynamic balance, and social domains of QOL.

Umiastowska and Kupczyk [41] examined factors differentiating the level of functional fitness of older adults (*n* = 509) assessed with FFFT, and they confirm a higher level of functional fitness among active older adults, both women, and men in comparison with the American standards. According to the age, it was observed that both Polish women and men achieved moderate results, exceeding the upper limit of the range in American standards for the following tests: arm curl, chair sit-and-reach, and 8-ft up and go. In the back scratch test, Polish seniors scored better. In the chair stand and two-minute step tests, the mean scores of the Polish respondents reached the upper limit of the range of standards in American studies.

Ihász et al. [54] aimed to assess the relationship between self-reported HRQOL and physical fitness (FFFT) in community-dwelling older females, including anthropometric parameters and body composition variables. The findings of this cross-sectional study of physical fitness and self-assessment of QOL in the group of older women showed that levels of physical fitness reflecting aerobic ability and muscle strength were significantly lower in the oldest group compared to the younger ones. The relationship between physical fitness and QOL was moderately and positively associated with physical functioning, as well as role limitations caused by physical and vitality problems. The authors call for action in research to determine the impact of an active lifestyle on functional performance (balance and resistance exercises) and increase the number of components to be assessed and conduct a longitudinal and interventional study.

Many researchers indicate age as one of the crucial predictors affecting QOL [55, 56]. In our study, in the women’s group, age proved to be an essential factor affecting the assessment in all domains and self-assessment of health. The analysis unequivocally showed negative relationships, namely, the older the woman, the lower the QOL score. The men’s group showed significant negative relationships between age and the psychological, social, and environmental spheres.

The evidence provided by Vagetti et al. [57] in their systematic review pointed out that the promotion of physical activity in the elderly population can have an impact exceeding the functional abilities and mental health because it involves a positive perception of the overall QOL. However, these findings also showed that physical activity might not be related to certain areas of QOL (e.g., sensory performance); therefore, further studies are needed.

To summarize, due to numerous factors influencing the QOL of people aged 80 and over, its assessment is problematic. Systematic physical activity allowing to maintain physical performance, independence, and autonomy, are factors influencing the enhancement of QOL, as demonstrated by the literature on the subject and the present study results. However, in QOL studies of elderly people, it should be remembered that the physical and mental health of these people is the result of life experience and the current situation, which is determined by many psychosocial and spiritual factors.

### Study limitations

This study has some potential methodological limitations. First of all, the cross-sectional protocol of this study makes it difficult to provide a clear interpretation of the relationship between the physical fitness parameters and QOL of the study participants. Secondly, the surveyed population is limited to quite a small number of participants (*n* = 100) living in the south-western regions of Poland, which makes it impossible to generalize the results for the entire population of Polish older adults. And the last but not least, the different group sizes concerning men and women were recruited in the present study. There is a need to conduct a prospective study on this important subject, including multicenter research. Moreover, further studies should also consider including more study outcomes such as anthropometric parameters (e.g., waist, arm, tight, and calf circumferences, waist-to-height ratio, arm fat area, skinfold thickness, and muscle thickness) and body composition parameters (fat percent, fat-free mass, visceral fat, bone mass, total body water, basal metabolic rate, and metabolic age). Also, it should be noticed that the age range is 80–83 and, thus, the results are not directly comparable to the studies where the participants we aged below 80 years old.

### Research directions

Undoubtedly, physical fitness is associated with QOL in older adults. Our findings emphasize the importance of maintaining good physical condition and its relationship with QOL levels. Because of the slightly lower physical fitness, women are exposed to reduced QOL. Therefore, further research should consider developing global standards and strategies for activity programs aimed at maintaining good physical condition, which could effectively improve overall QOL, especially in women. It seems necessary to promote the beneficial effects of health training on the organism through promotion and information programs for people coming into the autumn of life. People over 50 years of age should be aware that fitness and age-appropriate physical activity is the best way to maintain health, longevity, and well-being and encourage older adults to participate in physical activity programs [58]. Such programs, e.g., after 1 year of follow-up, appear to increase daily physical activity levels in older adults [59, 60].

## Conclusions

Community-dwelling older adults aged 80–93 years in Poland present a good level of QOL, and the higher score was obtained in men. Also, men presented better physical fitness, showed a higher level of independence in daily activities, and assessed better their own QOL than women. Physical, psychological, and environmental domains are important in the QOL assessment of older adults. The positive assessment of older men and women in terms of QOL is associated with the level of their physical fitness, in particular: mobility endurance, upper and lower body strength, balance, coordination, and speed.

## Data Availability

The datasets used and/or analyzed during the current study are available from the corresponding author on reasonable request.

## Appendix

**Table 7:** English translation of an original survey questionnaire designed by the authors including 40 questions in terms of biometric data, anthropometric characteristics, family and environmental situation, nutritional and lifestyle behaviors

No.	Item	Answer		
1	Name and surname			
2	Anthropometrics	Body height [cm]: _ _ _ _	Weight [kg]: _ _ _ _	
3	Birthday	Age [years]: _ _ _ _	Date of birth: _ _ / _ _ / _ _ _ _	
4	Sex [W/M]			
5	Marital status			
6	Lonely (since when?)			
7	Number of children			
8	Number of siblings			
9	At what age your siblings died?			
10	Siblings died of the causes	Natural:	Unnatural (accident, war):	
11	At what age your parents died?	Mother:	Father:	
12	Father died of the causes	Natural:	Unnatural (accident, war):	
13	Learned profession			
1.1.1.14	Level of education	a) Basic		
		b) Vocational		
		c) College		
		d) Higher		
15	The character of professional work	Mental	Physical	
16	Place of residence	City	Village	
1.1.1.1.17	Cigarettes smoking	a) I don’t smoke and I haven’t smoked		
		b) I’ve been smoking, I don’t currently smoke		
		c) I smoke 1–10 cigarettes a day		
		d) I smoke 10–20 cigarettes a day		
		e) I smoke more than 20 cigarettes a day		
1.1.18	Alcohol consumption	a) I do not drink alcohol (abstinence)		
		b) I consume alcohol on occasion		
		c) I consume alcohol frequently (once a week and more often)		
19	Living conditions	Good	Average	Bad
20	Material status	Sufficient	Insufficient	
1.1.1.21	How many main meals do you eat during the day?	a) Two or three		
		b) More than three		
		c) Only one		
		d) Very variously		
1.1.22	How many times a day do you snack between meals (e.g., cookies, candy bars, bread, meat, etc.)?	a) Not at all		
		b) One or two		
		c) Three or more		
1.1.1.23	How often do you eat red meat, such as beef, lamb, pork and their preserves (sausages, cold cuts, and pâtés)?	a) Twice a week or less frequently		
		b) Three or four times a week		
		c) Almost every day		
		d) Not at all		
1.1.24	How often do you eat fresh fruit and vegetables (raw or cooked)?	a) Twice a day and more often		
		b) Almost every day		
		c) Less than four times a week		
1.1.1.25	How often do you eat products such as brown, unshelled rice, thick groats, whole meal bread, oatmeal?	a) At least once a day		
		b) 3–6 times a week		
		c) Less than three times a week		
		d) Not at all		
1.1.1.26	How many times a week do you eat fried food?	a) Almost every day		
		b) About three times a week		
		c) Once a week or less frequently		
		d) Not at all		
1.1.27	Do you eat fast-food (hot dogs, hamburgers, pizzas), salty and fatty snacks (chips, sausages, crackers)?	a) Yes		
		b) Sometimes		
		c) Not at all		
1.1.28	How often do you eat fish?	a) Twice a week or more often		
		b) Less than once a week		
		c) Not at all		
1.1.1.29	How often do you eat butter, drink whole fat milk or use cream?	a) 3–4 times a day		
		b) Once a day		
		c) Several times a week or less frequently		
		d) I avoid fatty additives		
1.1.1.30	How many cups (glasses, mugs) of coffee do you drink a day?	a) Two or less		
		b) Three-four		
		c) More than five		
		d) Not at all		
1.1.1.31	How many glasses (cups) of sweetened drinks do you drink a day?	a) Two or less		
		b) Three-four		
		c) More than five		
		d) Not at all		
1.1.1.32	Do you often eat sweets (e.g., chocolate, candies, cakes)?	a) Every day or almost every day		
		b) About three times a week		
		c) Once a week or less frequently		
		d) Not at all		
1.1.33	How many liquids do you drink a day (water, tea, soup)?	a) About 3–6 glasses		
		b) About 6–10 glasses		
		c) More than 10 glasses		
1.1.34	Do you sometimes eat late at night?	a) Yes		
		b) Sometimes		
		c) No		
1.1.35	Do you sometimes eat in a hurry (e.g., breakfast)?	a) Yes		
		b) Sometimes		
		c) No		
1.1.1.1.36	How often do you check your weight?	a) Everyday		
		b) Once a week		
		c) Once a month		
		d) Once every six months		
		e) Not at all		
1.1.1.1.1.1.37	What kind of products did you eat as a child?	a) Meat		
		b) Milk, butter, cream		
		c) Breads		
		d) Sweets		
		e) Fruit		
		f) Vegetables		
		g) Fish		
1.1.1.1.1.1.38	Assign the following products to the individual floors of the health pyramid for seniors:	a) Meat, cold cuts, dairy products		
		b) Liquid, water, tea		
		c) Carbohydrates (bread, cereals, rice)		
		d) Fats, sweets, red meat		
		e) Vegetables and sweets		
		f) I don’t know what it is		

39	What diseases do you suffer from?			
1.1.1.40	Fill the values of the following tests:	a) Glucose level: _ _ _		
		b) Total cholesterol level: _ _ _		
		c) Blood pressure: _ _ _ / _ _		
		d) Hypertension (yes/no): _ _		

## Abbreviations

HRQOL: Health-related quality of life
QOL: Quality of life
WHOQOL-BREF: World health organization QOL, short form
FFFT: Fullerton functional fitness test
WHO: World health organization
STROBE: Strengthening the Reporting of Observational studies in epidemiology guidelines
